# Exploring the nature of the gender-congruency effect: implicit gender activation and social bias

**DOI:** 10.3389/fpsyg.2023.1160836

**Published:** 2023-05-23

**Authors:** Alba Casado, Ana Rita Sá-Leite, Francesca Pesciarelli, Daniela Paolieri

**Affiliations:** ^1^Mind, Brain, and Behavior Research Center, Department of Experimental Psychology, University of Granada, Granada, Spain; ^2^Department of Experimental Psychology, University of Granada, Granada, Spain; ^3^Department of Social Psychology, Basic Psychology and Methodology, University of Santiago de Compostela, A Coruña, Spain; ^4^Institut für Romanische Sprachen und Literaturen, Goethe University Frankfurt, Frankfurt, Germany; ^5^Department of Biomedical, Metabolic and Neural Sciences, University of Modena and Reggio Emilia, Modena, Italy

**Keywords:** gender-congruency effect, gender-priming, grammatical gender activation, gender stereotype, gender identity

## Abstract

The aim of the study was to explore the nature of the gender-congruency effect, characterized by a facilitation on the processing of congruent words in grammatical gender. Moreover, we explored whether resemblances between gender identities and gender attitudes with grammatical gender modulated lexical processing. We designed a gender-priming paradigm in Spanish, in which participants decided the gender of a masculine or feminine pronoun preceded by three different primes: biological gender nouns (mapping biological sex), stereotypical nouns (mapping biological and stereotypical information), and epicene nouns (arbitrary gender assignment). We found faster processing of gender congruent pronouns independently of the type of prime, showing that the grammatical gender feature is active even when processing bare nouns that are not conceptually related to gender. This indicates that the gender-congruency effect is driven by the activation of the gender information at the lexical level, which is transferred to the semantic level. Interestingly, the results showed an asymmetry for epicene primes: the gender-congruency effect was smaller for epicene primes when preceding the feminine pronoun, probably driven by the grammatical rule of the masculine being the generic gender. Furthermore, we found that masculine oriented attitudes can bias language processing diminishing the activation of feminine gender, which ultimately could overshadow the female figure.

## 1. Introduction

Grammatical gender is an intrinsic feature of nouns that in Romance languages distinguishes between masculine and feminine values (Corbett, 1991). Arbitrary words are those that are assigned grammatical gender arbitrarily and not based on semantic features of the noun, as is the case with nouns with human and animal referent. Thus, in Spanish, the gender distinction in inanimate nouns exclusively marks the declensional class (arbitrary gender) (Comrie, 1999), like “casa” FEM–*house*, but in most of animate nouns like “hermano” MAS–*brother*, it carries a semantic connotation related to the biological sex (natural or biological gender). The high correlation between the grammatical gender values (in principle an abstract grammatical feature) and biological sex in animate nouns favors the transference of the gender feature from a pure grammatical gender level to the lexical-semantic one (Sera et al., 1994, 2002; Vigliocco et al., 2005; Bender et al., 2018; Samuel et al., 2019; Casado et al., 2021). In this sense, grammatical gender may even influence the conceptual representations in languages with a formal gender system where the referents have a grammatical gender distinction [see a recent review by Samuel et al. (2019)],^1^ even when the grammatical gender assignment is purely arbitrary.

Previous research showed that in Romance languages grammatical gender is not merely a syntactic feature exclusively processed in noun phrase contexts for the sake of agreement, but a lexical property automatically activated (Cole and Segui, 1994; Bates et al., 1995; Cubelli et al., 2005; Alario et al., 2008; Paolieri et al., 2010; De Martino et al., 2011; Paolieri et al., 2011; Casado et al., 2018, 2021; Sá-Leite et al., 2021; see also Wang et al. (2018), for a similar lexico-syntactic classifier feature; and Sá-Leite et al., 2019 for a review). For instance, Paolieri et al. (2011) using a picture-word interference paradigm showed that naming pictures that were gender congruent with the grammatical gender of a superimposed distractor word was harder compared to when the gender of the distractor was incongruent, ultimately showing that during bare noun production, grammatical gender is automatically activated. Moreover, in a series of studies, Casado et al. (2018, 2021) showed that the grammatical gender of arbitrary words is primed by the sex of the speaker; when there is a match between the sex of the speaker (e.g., female) and the grammatical gender assignment (e.g., feminine), the words are processed faster in contrast to when there is a mismatch (e.g., female speaker–masculine word), indicating that grammatical gender is automatically activated during bare noun comprehension and suggesting a relation between grammatical gender and sex, which may directly impact lexical processing. All in all, the relationship between the grammatical and semantic information about gender is quite complex, such that grammatical gender and semantic information interact in word processing to influence integration of the noun into the sentence (e.g., Friederici and Jacobsen, 1999; Hagoort and Brown, 1999; Wicha et al., 2004). Note that grammatical gender information not only maps the biological sex distinction, but it can also bias the connotational meaning of some words in terms of gender stereotypes, that is, overgeneralized assumptions related to the behavior toward or preference for objects by males and females (e.g., males are tough; females are delicate). Stereotypes are particular to each culture and each person because they depend on the personal experience with the world (Allan, 2007; Segel and Boroditsky, 2011; Nicoladis and Foursha-Stevenson, 2012; Beller et al., 2015; Bender et al., 2016, 2018). Despite being part of the connotative meaning, stereotypical information is activated at very early stages of processing (Oakhill et al., 2005; Wang et al., 2017).

Siyanova-Chanturia et al. (2012) and later Pesciarelli et al. (2019) studied the automatic activation of gender stereotypes conveyed by single words in Italian, a Romance language. They designed a gender-priming paradigm where a prime word was followed by a grammatically masculine or feminine pronoun (“lui”–*he*, “lei”–*she*). As primes, they selected role nouns with a gender mark (e.g., “sorella” FEM–*sister*) or without a gender mark (e.g., “cantante”–*singer*). Moreover, words without a gender mark (bigender) could be stereotypically feminine (e.g., “insegnante”–*teacher*), masculine (“ingegnere”–*engineer*), or neutral (“cantante”–*singer*). The task of the participant consisted of deciding the grammatical gender of the pronoun, ignoring the prime. The results showed a gender-congruency effect: participants took longer to decide the gender of the target when incongruent with the gender of the prime. Importantly, participants took longer to decide the gender of the target when incongruent with the gender stereotype associated with bigender words. These results indicate that gender stereotypes influence language processing because the stereotype is activated and incorporated into the conceptual representation at very early stages of processing. Based on these results, the congruency effect could be interpreted as triggered by the activation of gender information not only at the grammatical but also at the semantic level (see Samuel et al., 2019).

To further explore the online processing of the stereotype, Siyanova-Chanturia et al. (2012) and Pesciarelli et al. (2019) measured the electrophysiological response evoked in the gender-priming paradigm. The authors focused on the N400 component, a negative-going deflection, in this case, peaking around 400 ms after stimulus onset, typically modulated by violations of semantic information and world knowledge (Van Petten and Kutas, 1990) and by lexical incongruence (Friederici and Jacobsen, 1999; Paolieri et al., 2020). Siyanova-Chanturia et al. (2012) and Pesciarelli et al. (2019) also examined the P300 component, an indicator of the subjective probability of eliciting an event.^2^ In both studies, they found that pronouns preceded by gender-incongruent primes elicited N400 components when the role nouns had a mark of grammatical gender and when the role nouns were associated with stereotypical gender (male or female) but grammatically unmarked (all words made reference to people). Interestingly, Siyanova-Chanturia et al. (2012) found an asymmetry with those role nouns associated with stereotypical gender. Replicating the results by Cacciari and Padovani (2007) and Irmen et al. (2010), they observed that the gender-congruency effect evoked by incongruent primes disappeared when processing feminine pronouns. This suggests that gender stereotypes are more restrictive for females than males, such that participants were more likely to accept females taking male roles (e.g., female firefighter) than males taking female roles (e.g., male nurse). Additionally, Siyanova-Chanturia found a P300 component elicited by pronouns preceded by gender-incongruent primes when the role nouns were marked by grammatical gender, restricted only to masculine pronouns in the study by Pesciarelli et al. (2019); that is, “cameriere” MAS (*waiter*)–lei (*she*) did not induce a P300 modulation. The lack of modulation of the P300 component with feminine pronouns indicates that the masculine form is often used to include the female referent as well, thus works as the generic gender (e.g., “pensionato” MAS–*pensioner* can also include the referent of female-pensioner). Altogether, the behavioral and ERP results from Siyanova-Chanturia et al. (2012) and Pesciarelli et al. (2019) indicate that the activation of gender information is automatic and induces the gender-congruency effect. Moreover, they revealed that the stereotypes are stronger for feminine role-names, and that the masculine form can work as the generic gender.

However, the nature of the gender-congruency effect is unclear. Given their stimuli selection, it is impossible to distinguish whether the gender-congruency effect is purely conceptual, or whether it is also influenced by the more abstract grammatical-lexical level of gender representation. As previously introduced, most animate nouns are gender marked and have a grammatical gender assignment congruent with the biological sex (e.g., “hermano” MAS–*brother*; “hermana” FE–*sister*). However, there are some exceptions like the epicene nouns (e.g., “tortuga” FE–*turtle*) which refer to animate entities but have an arbitrary gender assignment because their grammatical gender is not informative about the biological sex distinction (Vigliocco et al., 2005). By observing the processing of epicene nouns, we could explore if the nature of the gender-congruency effect is grammatical and not necessarily associated with the conceptual information about biological sex.

### 1.1. Current study

We designed a gender-priming experiment in Spanish similar to the one of Pesciarelli et al. (2019), in which we included gender marked nouns referring to animate entities, for which the gender-congruency effect should be stronger (Vigliocco et al., 2005). However, we not only selected role-nouns without an associated gender stereotype–neutral or biological gender (e.g., “alumno” MAS–*student*)–and role-nouns with an associated gender stereotype always congruent with the grammatical gender–stereotype (e.g., “enfermera” FEM–*nurse*) –, but also animals’ nouns arbitrarily gendered–epicene (e.g., “tortuga” FEM–*turtle*).

If the gender-congruency effect is evoked by the activation of the conceptual information related to biological gender, we should observe the gender-congruency effect in neutral and stereotypical primes, at least for masculine pronouns [following the gender asymmetry found by Cacciari and Padovani (2007), and Siyanova-Chanturia et al. (2012) for stereotypical primes and Pesciarelli et al. (2019), for neutral primes]. Moreover, the effect of gender congruency should be larger in stereotypical primes (at least for female role-names, e.g., “enfermera” FEM–*nurse*) compared with neutral primes (e.g., “alumno” MAS–*student*), since the activation of the conceptual representation of males and females would be active bidirectionally by the biological sex and by the connotational meaning as well. On the contrary, we should not observe the gender congruency effect in epicene primes, because gender here is not informative of the biological sex of the animal, so the conceptual representation associated with the biological sex should not be activated. In contrast, if the gender-congruency effect can be evoked by the activation of the lexical-grammatical information about gender, we should observe the gender congruency effect in neutral primes, in stereotypical primes, and also, in epicene primes. That is, despite gender not being informative of the biological sex of the referent in epicene nouns, the activation of grammatical gender values may induce the gender-congruency effect through competition based on grammatical-lexical information.

Additionally, we wanted to explore the role of the masculine form as the generic gender because the variability in lexical gender assignment moderates predictive gender agreement processing (Hopp, 2016). As previously introduced, Pesciarelli et al. (2019) only found the P300 component evoked when masculine marked primes preceded the feminine pronoun (“dottorando” MAS *PhD student*–she), indicating a probabilistic bias: it is more likely to find masculine words referring to females, but it is not that common to find feminine words referring to males (although this can be different in certain communities like LGTBIQ+). For instance, in Spanish it is common to use the word “chicos” MAS–*guys* to call the attention of a group of people, even when this group includes females. In fact, in Spanish the rule posits that the masculine form should be used as the generic gender.

However, little is known about the cognitive processing of this grammatical rule. Previous behavioral data indicate that there is a male bias when using generic forms [see Lindqvist et al. (2019) for evidence in Swedish and English], in particular when processing the masculine form as the generic (Stetie and Zunino, 2022) because the generic representation really depends on the stereotypicality of the role nouns. In the present study, we wanted to dig into this issue by including stereotypical role-nouns and neuter role-nouns. Moreover, we chose to present the plural form of primes (“chicos”) and targets to increase the chances to find the effects related to generic gender and stereotypicality in the behavioral results.

Importantly, we were also interested in exploring additional factors influencing the gender-congruency effect that may give us information regarding the possible relation between biological sex distinctions and gender identities, as well as grammatical and biological linguistic gender. Previous studies found that females were more sensible to the gender-congruency effect than males (Siyanova-Chanturia et al., 2012; Pesciarelli et al., 2019).^3^ Given that biological sex influenced differently the way males and females processed gendered words, in the present study we wanted to go a step forward and study the influence of not only the biological sex, but also the self-gender representation on the gender-congruency effect. In particular, we asked the participants to fill-in the Bem sex roles inventory (Bem, 1974) to explore whether gender diversity was driving the previously found biological sex modulation. The Bem sex roles inventory informs about different gender profiles (feminine, masculine, androgen, and indifferent) based on a Likert scale on how much people identify themselves with different adjectives. In line with Morgenroth and Ryan (2020), we wanted to include a non-binary and fluent vision of gender to avoid possible bias due to binary socialization in gender roles (Eagly and Wendy, 2017).

Besides, we wanted to explore whether the sexist behavior, in particular the degree of positive and/or negative feminine discrimination, influenced the gender-congruency effect, especially when processing the generic masculine. Previous research (Vergoossen et al., 2020) found some dimensions that capture the assumptions and beliefs underlying reasons against gender-fair language, among them sexism and cisgenderism. To detect whether benevolent sexism (e.g., women are weaker and therefore need protection from men) or hostile sexism (e.g., women are inferior to men and therefore hopeless) influenced the processing of the masculine form as the generic gender we considered the scores obtained through the Ambivalent sexist inventory (Cárdenas et al., 2010 based on Glick and Fiske, 2001). In the questionnaire, people answer how much they agree or disagree with stereotypical sexist information. From the answers two scores are derived: benevolent sexism, and hostile sexism.

Not only were we interested in the participants’ beliefs and behavior regarding sexism, but in the real language daily use. That is, whether, in fact, they use non-sexist language like alternative desinences for gender (e.g., -e, -x), double forms to visualize women (e.g., “alumnos y alumnas”— MAS and FE *students*), or the feminine as the generic when most of the group is female. To check the role of inclusive language use in gender processing, we asked the participants to fill-in the Sexist language questionnaire (adapted from Jiménez Rodrigo et al., 2011). In the questionnaire the participants answer how much they agree or disagree with some language use and situations. From the answers, a score of sexist language use is derived. All the questionnaires can be found in Appendix A; there the Spanish version of each of the questionnaires can be found in separate Excel sheets.

## 2. Materials and methods

### 2.1. Participants

We recruited 54 Spanish native speakers from the University of Granada. All of them were young adults with high educational level. All participants received monetary compensation for taking part in the study (5 euros) or university credits for the degree in Psychology.

From the initial sample of participants, we excluded 9 participants that had an accuracy score below 60%. The final sample was formed by 45 participants (34 females), average age 20.8 y.o. The study met the requirements and gained the approval of the Ethics Committee of the University of Granada concerning experimental studies with human subjects (UGR n.: 2702/CEIH/2022).

### 2.2. Material

As primes, we selected 120 Spanish plural nouns, half of them masculine and half of them feminine. From the 120 nouns we created 3 different subgroups: (1) 40 epicenes, that is, words referring to animals without explicit information about their biological gender (e.g., “gusanos” MAS—worms, “jirafas” FE–giraffes), (2) 40 stereotypical gender words marked for gender, that is, words referring to professions or roles typically associated with males or females (e.g., “barberos” MAS—barbers, “enfermeras” FE—nurses), and (3) 40 neutral words, that is, words referring to people marked for gender but not particularly associated with gender stereotypes (e.g., “suegros” MAS—fathers in law, “señoras” FE—ladies). The stereotypical words were selected based on the results of a questionnaire in which participants saw the masculine and feminine version of the role name [e.g., “enfermero” (nurse, MAS)/“enfermera” (nurse, FE)], and they decided from 1 to 7 how masculine or feminine this role was, being 1 “very feminine” and 7 “very masculine.” As fillers, we included 40 words with arbitrary gender assignment (e.g., “taladros” MAS—drills, “sillas” FE—chairs).

As targets we used the plural forms of the demonstrative pronouns referring to people (e.g., “estas”/“estos”—these). We selected demonstrative pronouns to be able to include the fillers (arbitrary gender words) without additional variations on grammatically concordances besides grammatical gender.

All the target nouns were controlled by lexical frequency as measured with Subtlex (Cuetos et al., 2012), the length (number of letters), orthographical neighbors, and phonological neighbors from EsPal database (Duchon et al., 2013) for each subgroup, see Table 1. A complete list of materials can be found in Appendix B.

**TABLE 1:** Words’ characteristics.

Gender type	Grammatical gender	Lexical frequency	t.test	Length	t.test	Orthographical neighbors	t.test	Phonological neighbors	t.test
Epicene	fem	1.81 (0.470)	*t*(36) = 1.33; *p* = 0.19	8.25 (1.37)	*t*(36) = −0.68; *p* = 0.50	1.6 (2.97)	*t*(35) = −0.18; *p* = 0.86	3.9 (4.47)	*t*(37) = 0.16; *p* = 0.87
	masc	1.58 (0.590)		8.6 (1.77)		1.8 (3.92)		3.65 (5.09)	
Biological	fem	1.81 (0.709)	*t*(37) = −0.31; *p* = 0.76	9.05 (2.48)	*t*(27) = 1.268; *p* = 0.22	2.6 (2.38)	*t*(35) = 1.03; *p* = 0.31	5.25 (4.14)	*t*(37) = 1.15; *p* = 0.25
	masc	1.89 (0.811)		8.25 (1.22)		1.9 (1.76)		3.8 (3.57)	
Stereotypical	fem	1.37 (0.630)	*t*(38) = −2.10; *p* = 0.04	9.1 (1.48)	*t*(34) = 1.55; *p* = 0.13	2.7 (2.39)	*t*(29) = 1.51; *p* = 0.14	4.7 (3.39)	*t*(37) = 0.14; *p* = 0.88
	masc	1.82 (0.684)		8.45 (1.07)		1.75 (1.34)		4.55 (2.96)	
		ANOVA	*F*(2) = 1.38; *p* = 0.26	ANOVA	*F*(2) = 0.44; *p* = 0.64	ANOVA	*F*(2) = 0.55; *p* = 0.58	ANOVA	*F*(2) = 0.52; *p* = 0.59

### 2.3. Procedure

The participants were tested in an isolated room where they were seated in front of a computer screen. They received written instructions on the computer; their task consisted of answering as quickly and accurately as possible whether the pronoun (target word — “estas”/“estos”) was masculine or feminine. They had two possible response buttons: “F” for feminine words, and “M” for masculine words. Before starting the task, the participants saw 10 practice trials to get used to the paradigm that were excluded from the analyses. Each trial followed the same structure: (1) fixation cross for 500 ms in the middle of the screen; (2) prime for 50 ms; (3) target for 1,500 ms or until the participant’s response; (4) black screen for 1,000 ms before the next trial. The procedure replicates the previous study by Pesciarelli et al. (2019).

We created two versions of the same experiment counterbalancing the congruency of the primes and targets. We first randomized the gender congruency for one version [e.g., “mesas” FE (*table*) — “estas” FE (*these*) = congruent], and from that, we created the opposite version [e.g., “mesas” FE (*table*) — “estos” MAS (*these*) = incongruent]. The participants were assigned to each of the versions randomly.

At the end of the main task, the participants were asked to fill-in some questionnaires: 1) Bem sex roles questionnaire (Bem, 1974), Stereotype questionnaire (to control for the perceived degree of stereotype for each of the stimuli presented in the main task), Sexist language questionnaire (adapted from Jiménez Rodrigo et al., 2011), and the Ambivalent sexist inventory (Cárdenas et al., 2010), see Appendix A for more detail; there the Spanish version of each of the questionnaires can be found in separate Excel sheets.

### 2.4. Statistical analyses

All analyses were performed using linear mixed-effect models, as implemented in the lme4 package (version 1.1-27.1; Bates et al., 2021) in R using participants and items (primes) as crossed random effects. We selected the response times (RTs) as the dependent variable. We filtered the data by accuracy (only including correct responses); we also filtered the outliers by excluding RTs above 1,000 ms, and 2.5 sd from the mean of each participant. In total, we excluded 6.02% of the data due to errors of accuracy, 1.03% of the data due to the 2.5 sd filter, and 0.99% excluded after filtering the RT above 1,200 ms. Due to the right skewed distribution of the data, we inverted the RT score (−450/RT).

We created a mixed-effect model to explore the role of the grammatical gender categories. In the model, we included as fixed effects the gender_type (epicene, stereotype, and neutral), the prime_gender (feminine, masculine), the target_gender (feminine, masculine), and the interactions between them. As control variables, we included the version (1, 2), the trial_number (1–160), the length of the words, and the lexical frequency. We transformed the continuous variables (trial_number, length, lexical frequency) to normalize the distribution by scaling the scores. We selected sum contrast for gender_type (baseline = neutral), prime_gender (feminine = −1; masculine = 1), target_gender (feminine = −1; masculine = 1), and version (1 = −1, 2 = 1).

We performed further analysis by adding to the main model additional variables separately; thus, new models were created to explore the role of the biological sex of the participants, the sex-role obtained in the Bem sex role questionnaire (Bem, 1974), the benevolent sexist behavior (Cárdenas et al., 2010), the hostile sexist behavior (Cárdenas et al., 2010), and the sexist language use [adapted from Jiménez Rodrigo et al. (2011)].

We fitted the maximal model first (Barr et al., 2013), and in case of non-convergence or singularities we simplified it following recommendations outlined in Bates et al. (2021). We considered as significant any fixed effect with t-statistic higher than 2. For simplification purposes, we present in the main text the Anova from the mixed-effect model. The regression outcomes are presented in Appendix C; there the outcomes of each of the models are shown in a separate Excel sheet (main analysis, further analyses including: biological sex, Bem sex role questionnaire, benevolent sexist behavior, hostile sexist behavior, and sexist language use).

## 3. Results

**FIGURE 1:**
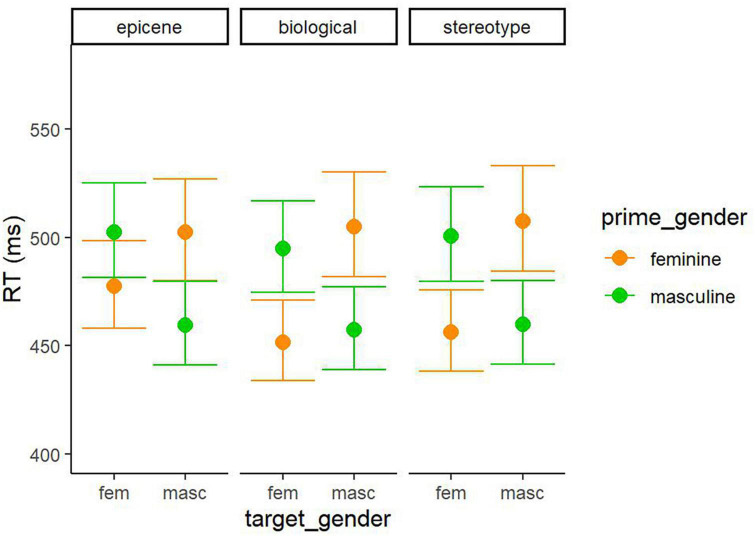
Model-based predictions of the gender-congruency effect for each of the gender types: epicene nouns (arbitrary gender assignment), biological gender nouns (mapping biological sex), and stereotypical nouns (mapping biological and stereotypical information).

The results showed a main effect of gender_type, so there were slower responses to targets preceded by epicene primes than to targets preceded by biological primes, and slower than targets preceded by stereotypical primes (see Table 2). Moreover, there was an interaction between the gender_type and target_gender. Feminine targets were processed slower when preceded by epicene primes compared to when preceded by stereotypical or biological primes. As predicted, there was an interaction between prime_gender and target_gender, revealing that when the prime was feminine, the feminine target was processed faster (mean = 463 ms) than the masculine target (mean = 505 ms). In the same line, when the prime was masculine, the masculine target was processed faster (mean = 459 ms) than the feminine target (mean = 499 ms). This interaction between the prime_gender and target_gender was modulated by the gender type. We can observe in Figure 1 that the congruency effect was smaller for the feminine target when preceded by epicene primes than when preceded by the stereotypical or neutral primes. No differences between gender types were found for the masculine target. See Table 3 for the effect sizes. Furthermore, there was a main effect of trial number, such as the experiment advance, the slower the responses became.

**TABLE 2:** ANOVA mixed-effect model.

	Sum sq	Mean sq	NumDF	DenDF	*F* value	Pr(>*F*)
Gender_type	0.2166	0.1083	2	4475.6	3.4686	0.03125*
Prime_gender	0.0451	0.0451	1	43.4	1.4446	0.23592
Target_gender	0.0023	0.0023	1	43.6	0.0723	0.78931
Version	0.0328	0.0328	1	42.8	1.0491	0.31146
Trial.s	0.5461	0.5461	1	4525.1	17.4912	0.0001***
Length.s	0.0628	0.0628	1	4491.4	2.0101	0.15632
Freq.s	0.0444	0.0444	1	4501.7	1.4234	0.2329
Gender_type:prime_gender	0.0615	0.0308	2	4478.2	0.9849	0.37354
Gender_type:target_gender	0.2547	0.1274	2	4479.1	4.0794	0.01698*
Prime_gender:target_gender	7.544	7.544	1	4479.7	241.6189	0.0001***
Gender_type:prime_gender:target_gender	0.1589	0.0795	2	4476.9	2.5451	0.07858

*p < 0.05, **p < 0.01, ***p < 0.001.

**TABLE 3:** Mean and effect sizes.

Epicene	FEM target	MAS target
FEM prime	477.200	502.232
MAS prime	502.232	459.653
Pairwise	*z* = −3.602; *p* = 0.0003	*z* = 6.416; *p* = 0.0001
Effect size	−0.265	0.471
Biological	FEM target	MAS target
FEM prime	456.389	484.392
MAS prime	500.556	441.176
Pairwise	*z* = −6.462; *p* = 0.0001	*z* = 6.854; *p* = 0.0001
Effect size	−0.494	0.520
Stereotypical	FEM target	MAS target
FEM prime	451.807	505.051
MAS prime	495.050	457.317
Pairwise	*z* = −6.231; *p* = 0.0001	*z* = 6.772; *p* = 0.0001
Effect size	−0.491	0.524

### 3.1. Sex of the participant

**FIGURE 2:**
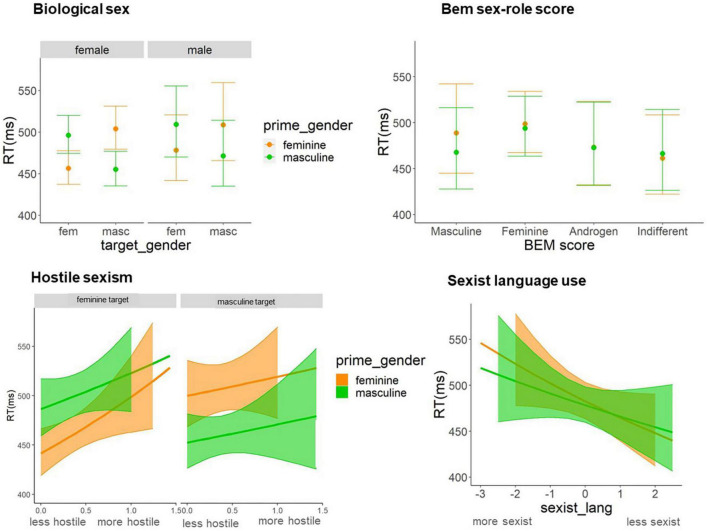
At the **left** side, model-based predictions of the gender-congruency effect modulated by the biological sex of the participants (female, male) and the scores in hostile sexism (less hostile, more hostile). At the **right** side, model-based predictions of the interaction between the prime gender and the Bem sex-role score (androgen, feminine, non-differentiated, masculine) and the sexist language use (more sexist, less sexist).

There was an interaction between the sex of the participant and the target gender, and prime gender. Both males and females experienced the congruency effect, being the effect sizes bigger for females than males. See Figure 2 and Appendix D.

### 3.2. Bem sex role questionnaire

There was an interaction between the score in the Bem sex role questionnaire and the prime gender: those participants classified as masculine processed faster the targets preceded by masculine primes (mean = 467 ms) than preceded by feminine primes (mean = 489 ms), *z* = 3.308; *p* = 0.0009. The rest of contrasts (androgen, feminine, indifferent) were not significant (see Figure 2 and Appendix D).

### 3.3. Ambivalent sexism

The scale of benevolent sexism did not interact with any factor.

The scale of hostile sexism interacted with the target gender: the higher the hostile sexism, the faster the responses to the targets when preceded by masculine primes than when preceded by feminine primes. Furthermore, these two factors interacted with the prime gender, showing that the gender congruency effect was invariant for the masculine targets, while for the feminine targets it only appeared for those participants with lower scores in hostile sexism. See Figure 2 and Appendix D.

### 3.4. Sexist language questionnaire

There was an interaction between the score in the sexist language questionnaire and the prime gender: the more sexist the language was, the faster the response to the targets when preceded by masculine primes than when preceded by feminine primes. See Figure 2 and Appendix D.

## 4. Discussion

In this work, we aimed to study the relationship between grammatical and biological gender by exploring the differential weight that conceptual and purely grammatical features may have on the triggering of the so-called gender-congruency effect. We designed a gender-priming paradigm in which participants decided the gender of a masculine or feminine pronoun preceded by primes of the same or different grammatical gender values. The primes were neutral role-nouns (mapping biological sex), stereotypical role-nouns (mapping biological and stereotypical information), and epicene nouns (arbitrary gender assignment).

Given that previous studies found an interaction between biological sex and grammatical gender (e.g., Casado et al., 2018, 2021; Pesciarelli et al., 2019), biological sex and gender identities may impact the way gender values are processed. For this reason, we decided to explore whether the gender congruency effect may be influenced by social factors. On the one hand, we explored whether Spanish speakers conceptualize the masculine gender as the generic gender value (i.e., whether it cognitively represents both male and female referents) and if so, whether it is impacted by the associated gender stereotype. To do so, all primes and targets were presented in plural forms. On the other hand, we explored the role of biological sex, sex-role, sexist behavior, and the use of sexist language on the gender-congruency effect. We did so by applying a series of questionnaires and performing further analyses to observe the influence of these factors on the gender-congruency effect.

The results revealed a gender-congruency effect for all prime types, including epicene nouns. Given that the grammatical gender assignment of epicene nouns is arbitrary and does not correlate with the biological sex of animate entities, the results indicate that the gender-congruency effect is driven by the activation of the grammatical gender feature even when processing bare nouns that are not conceptually related to gender.^4^ Furthermore, we found similar effect sizes of the gender-congruency effect for biological (neutral) and stereotypical primes, indicating that the extra information about the biological gender coming from the biological gender (semantic) and from the associated stereotype (connotational) did not increase the gender-congruency effect. Altogether, it seems that the nature of the gender-congruency effect may not be exclusively driven by the activation of conceptual information related to biological gender, but at least in part may be driven by the activation of the gender information at the lexical-grammatical level, that is transferred to the semantic level especially for nouns mapping gender conceptually (i.e., the neutral and stereotypical role-nouns).

Interestingly, the results showed an asymmetry for epicene primes, since the gender-congruency effect was smaller for epicene primes when preceding feminine targets, based on the smaller difference in RT when the feminine target was followed by a feminine prime vs. when followed by a masculine prime, compared with biological and stereotypical words [i.e., it is similarly demanding to process “tortugas” FE (*turtles*)–“estas” FE and “canguros” MAS (*kangaroos*)–“estas” FE]. This asymmetry follows a similar pattern to the one described by previous studies with stereotypical primes (Cacciari and Padovani, 2007; Irmen et al., 2010; Siyanova-Chanturia et al., 2012) and with gender marked primes without an associated gender stereotype (Pesciarelli et al., 2019). Based on previous literature, we could speculate that the reduced gender-congruency effect for feminine pronouns may be caused, at least partially, by the grammatical rule of the masculine being the generic gender, since the female referent can be included in the masculine form (e.g., the Spanish word “profesores” MAS— *professors* can include both male and female referent, while the Spanish word “profesoras” FE — *female professors* only includes female referents). Probably, the generic male rule was applied to animal nouns such that readers activated both male and female referents when processing words like “canguros” MAS (kangaroos), but they only activated the female referent when processing the word “tortuga” FE (turtle). In a similar vein, we could speculate that the reduced gender-congruency effect for feminine pronouns may be caused, at least partially, by the grammatical rule of the feminine being the “marked” gender (the one carrying the greater amount of gender information, see Prado, 1982; Harris, 1991; Beatty-Martínez and Dussias, 2019). This being so, the specified nature of the feminine prime noun would cause more disruption in the incongruent condition with the masculine pronoun, than a generic masculine would cause with the feminine pronoun, as the masculine generic is less specified for gender. Still, it is singular to find the asymmetry exclusively on epicene nouns, and not on neuter and stereotypical role-names, as previously found. This could be partially explained by the selection of the target word. Siyanova-Chanturia et al. (2012) and Pesciarelli et al. (2019) used the personal pronoun of the 3rd person in singular (“lei” -*he*, “lei”–*she*), which only references animate entities; in contrast, we selected the demonstrative pronoun in the 3rd person of the singular (“este”/“esta”–*this*), which can reference animate and inanimate entities. This may have caused a decrease in the effect of the masculine as the generic. In addition, we must acknowledge that in animals, the biological sex is less salient than in people, and consequently, the generic gender rule is easier to apply. Another possibility is that the absence of semantic meaning of the gender assignment facilitates the application of syntactic rules in epicene nouns compared with role-names. All in all, comparing epicenes with biological and stereotypical gender words showed that the association of gender with biological sex is mainly apparent in nouns referring to humans. Thus, in biological and stereotypical gender words (which represents humans) grammatical gender and biological sex mostly coincide so the masculine as the generic rule is not automatically applied. Finally, it is important to consider that here we explored behavioral data and more subtle differences may be captured with more sensitive techniques like ERPs, especially through the analysis of N400 components. Thus, further research is needed to better explain the asymmetry in the gender-congruency effect in gender arbitrary words, in contrast to stereotypically and biologically gendered words.

In line with our aims, one of the factors that could be influencing the gender-congruency asymmetry may be the participants’ gender representation, and their sexist attitudes, so we performed further analyses including as fixed factors the outcomes of the questionnaires we applied. To start with, we explored the role of the biological sex of the participants. The results revealed, in line with Pesciarelli et al. (2019) that females were more sensitive to the gender-congruency effect in contrast to males. These gender differences have been attributed to a more pronounced sensitivity of females toward grammatical violations, reflecting better language-related skills than males (Siegel, 2001). Apart from the pure biological sex, we wondered whether the gender identity or sex-role representation were underlying this modulation.

To this aim, we included as a factor in the analysis the sex role profiles (masculine, feminine, androgen, or indifferent) derived from Bem sex role inventory (Bem, 1974). The results revealed differences between the role profiles: in contrast to the rest of profiles, the more masculine participants processed faster the masculine prime information, indicating that there was a facilitation to process masculine words. In short, the participants with masculine profiles biased the processing of words matching their own sex-role representation. This may indicate indirectly that people with masculine sex-role use more frequently the masculine forms, and probably, they will be more sensitive to the use of the masculine as the generic. In fact, an exploratory analysis showed that in contrast to the rest of the groups, the more masculine participants did not experience facilitation when processing feminine targets preceded by feminine primes; moreover, this difference was not exclusive for epicene nouns, but also for stereotypical nouns (see Appendix D, sheet “Bem-means”). Despite the novelty, these results should be taken cautiously, and further research is needed to confirm these findings given the small number of participants in each sex-role group.

Additionally, we wondered whether sexists’ beliefs and behavior could influence the gender-congruency effect. We used the Ambivalent sexism questionnaire (Cárdenas et al., 2010) to differentiate between people positively discriminating women (benevolent sexism), and negatively discriminating women (hostile sexism). The results showed no effects of the benevolent sexism scale. In contrast, the hostile sexism scale did play a role. The hostile sexism scale did not influence the gender-congruency effect with masculine targets but it influenced the effect with feminine targets. More specifically, as expected, less hostile participants showed the effect of gender-congruency for feminine targets, but the effect was absent for the most hostile participants. These results indicate that the participants with hostile attitudes toward females do not experience facilitation associated with processing feminine words. Thus, it seems like participants have a shallower processing of the feminine concept in general, to the extent that it lowers the activation of the feminine grammatical gender at the lexical level. This novel finding indicates that sexist beliefs and attitudes can bias even the processing of grammatical rules. Future studies should further dig into the transference of sexist beliefs to general language processing.

Lastly, we were interested in exploring whether the realistic use of inclusive language influenced the gender-congruency effect, especially the processing of the masculine as the generic gender. We implemented the Sexist language questionnaire [adapted from Jiménez Rodrigo et al. (2011)], which determines the participants’ beliefs about the use of inclusive language. The results did not show an impact of the beliefs regarding the use of inclusive language on the gender-congruency effect itself, but they did show its impact on the processing of gender values. In particular, in contrast to the linguistically inclusive participants (who did not have a differential process of masculine or feminine primes), the participants against the inclusive language policies were biased such as they processed the masculine prime information faster, indicating that there was a facilitation to process masculine words. This finding seems to align with the results of the Bem sex-role inventory, which suggested that participants with male sex roles showed biased processing of the nouns matching their own sex-role representation.

In sum, in this work we have found evidence supporting the idea that the gender-congruency effect is driven by the activation of the gender information at the lexical level, which is transferred to the semantic level. Thus, the gender-congruency obtained for nouns mapping biological sex and stereotypical information in gender-priming paradigms may have not only a conceptual but also a lexical basis. Importantly, we have found an asymmetry between gender values that may be explained by the grammatical rule of the masculine being the generic value–yet more research is necessary to understand why this asymmetry was absent for nouns mapping biological gender but present for animate nouns with abstract gender assignment. When further exploring the possible consequences of the parallelism between grammatical gender and biological sex/gender identities, the results of a series of questionnaires suggested that such parallelism may be indeed impacting lexical processing at the level of grammatical gender activation. More specifically, we found that the masculine oriented behaviors benefited the processing of masculine information and negatively affected the processing of feminine information, which ultimately could influence the visibility of female figures. In contrast, non-masculine oriented behaviors seemed to promote similar processing of masculine and feminine information. Altogether, the questionnaires’ results showed that masculine oriented attitudes can bias language processing, and therefore promoting alternative attitudes could be beneficial for eliminating cognitive bias in language processing.

## Data availability statement

The original contributions presented in the study are publicly available. This data can be found here: https://osf.io/rh3sn/.

## Ethics statement

The studies involving human participants were reviewed and approved by UGR no.: 2702/CEIH/2022. The patients/participants provided their written informed consent to participate in this study.

## Author contributions

DP and FP: conceptualization. AC: data curation, formal analysis, validation, visualization, and writing—original draft. DP: funding acquisition, project administration, resources, and supervision. AS-L: investigation. DP and AS-L: methodology. AC, AS-L, and FP: software. AC, DP, AS-L, and FP: writing—review and editing. All authors contributed to the article and approved the submitted version.

## Footnotes

1 The transference from grammatical to conceptual gender is more robust in adults performing linguistic tasks with explicit gender/sex activation, in formal gender languages (especially with two gender categories), and with single words referencing animate referents.

2 The P300 component is typically observed in priming designs, when an immediate response is required (Barber and Carreiras, 2005).

3 In terms of stereotype, Proverbio et al. (2018) found a different pattern of gender differences in the processing of gender stereotypical information: males were found to have more bias than females, especially toward male characters.

4 Besides grammatical gender, there are other aspects of grammar like the Differential Object Marking for which the animacy of the referent (animals vs. humans) is relevant (Sagarra et al., 2020).
